# Incidence of Alpha-Gal IgE Sensitization in 3000 Military Personnel, Assessing Sex, Race, Installation, and Occupational Impacts

**DOI:** 10.3390/jcm13237162

**Published:** 2024-11-26

**Authors:** Susan J. Ching, Apryl Susi, Samuel M. Ailsworth, Lisa J. Workman, Thomas A. E. Platts-Mills, Jeffrey M. Wilson, Cade M. Nylund

**Affiliations:** 1 Department of Pediatrics, Uniformed Services University of the Health Sciences, Bethesda, MD 20814, USA; susan.j.ching.do.mph@gmail.com (S.J.C.);; 2 Henry M. Jackson Foundation for the Advancement of Military Medicine, Bethesda, MD 20814, USA; 3 Division of Allergy and Clinical Immunology, Department of Medicine, University of Virginia, Charlottesville, VA 22903, USA

**Keywords:** alpha-gal, alpha-gal syndrome, galactose-alpha-1,3-galactose, mammalian meat allergy, *Amblyomma americanum*

## Abstract

**Background/Objectives**: IgE to galactose-alpha-1,3-galactose (alpha-gal) is associated with *Amblyomma americanum* (lone star tick) bites, accounting for the regional distribution of the alpha-gal syndrome (AGS). Longitudinal studies describing risk factors for incident alpha-gal sensitization are lacking. The objective of this project was to assess the incidence of alpha-gal IgE seroconversion and identify associated demographic, occupational, and geographical risk factors among US military personnel. **Methods**: Samples from the Department of Defense Serum Repository were evaluated at two time points at least 3 years apart. In total, 3000 service members stationed at 10 military installations within the *A. americanum* tick range were included. Installation, sex, race and ethnicity, rank, military occupation, and branch of service were evaluated. Alpha-gal IgE seroconversion was defined as a change from <0.1 kU/L) to ≥0.1 kU/L. **Results**: Among the 2821 personnel who were alpha-gal IgE-negative at baseline, 138 (4.9%) seroconverted over a mean interval of 3.4 years. Seroconversion was more frequent in males (5.5% vs. 1.9%), White individuals (6.6% vs. 1.0% in Black people and 1.5% in Hispanics), and individuals in occupations with higher presumed outdoor exposure (e.g., infantry/law enforcement: 12.7% vs. administrative: 1.2%). Differences were not significant between sexes when accounting for military installation/occupation, but differences in race and ethnicity remained significant. **Conclusions**: This study demonstrates that alpha-gal IgE seroconversion is occurring within the *A. americanum* tick range and is associated with White race and ethnicity, and occupations with higher outdoor exposure. Further research is needed to elucidate the influence of race and ethnicity on alpha-gal sensitization and develop effective prevention and treatment strategies for AGS.

## 1. Introduction

Food allergies are increasingly appreciated as a cause of morbidity and mortality around the globe [1,2]. Immunoglobulin E (IgE) antibodies specific for the oligosaccharide galactose-alpha-1,3-galactose (alpha-gal) are an important cause of mammalian meat allergy, now commonly referred to as the “alpha-gal syndrome” (AGS) [3]. AGS may manifest similar to other food allergies with urticaria, angioedema, and anaphylaxis; however, it is unique in that symptoms are most often delayed by 3–6 h after the ingestion of mammalian meat [4,5,6]. Tick bites, particularly from the tick *Amblyomma americanum* (lone star tick), are suspected to be the major cause of alpha-gal sensitization in the United States (US), contributing to regional variations in AGS prevalence, notably in southeastern regions of the US [7,8,9,10,11,12]. Some cross-sectional studies have suggested differences in AGS prevalence based on sex and race and ethnicity, but there is a lack of longitudinal studies evaluating how tick bites and other factors impact the risk of developing IgE to alpha-gal [9,13].

Military members, like the overall US population, have been noted to have an increasing rate of food allergies compared to previous surveillance [14]. For AGS in particular, there are several factors that may place military members at risk. More than 55 percent of all US military members are assigned to an installation within the range of *A. americanum* and outdoor occupations may place military members at risk of tick-borne disease [15,16,17]. Other studies of both military and US populations noted an increased risk of acquiring tick-borne disease secondary to occupational or outdoor activities [18].

Here, we sought to investigate alpha-gal IgE sensitization in military personnel assigned to select installations within the range of *A. americanum*, specifically focusing on individuals who converted from a negative to a positive alpha-gal IgE. We also evaluated the impact of sex, race and ethnicity, geography, and military occupation on the incidence of alpha-gal sensitization.

## 2. Materials and Methods

**Figure 1:**
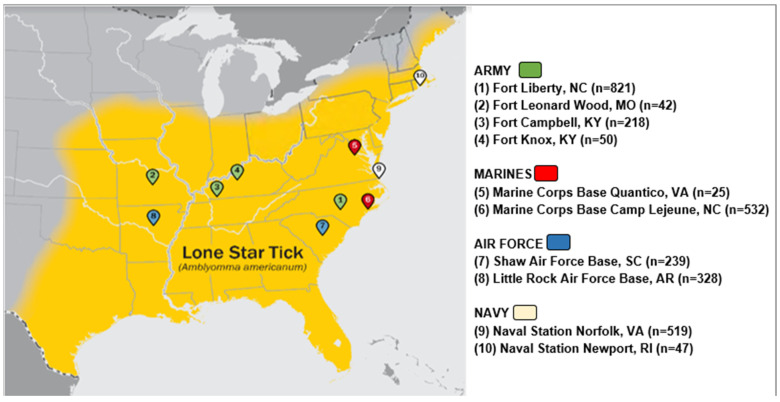
Location of military installations in relation to known lone star tick distribution at the time of the blood samples.

Using banked sera from the Department of Defense Serum Repository (DoDSR), this longitudinal serological study (a repeated cross-sectional analysis) was performed assessing IgE levels to alpha-gal in US active-duty military personnel. The DoDSR collects excess serum from military members completing periodic and mandatory HIV screening and is the largest bank of human serum in the world [19,20]. Study participants were included if they were active-duty service members in the U.S. Army, Air Force, Navy, or Marine Corps whose first assignment following the completion of basic training was at one of ten military installations. The installations for sampling were selected based on the reported range of *A*. *americanum* at the time of serum draw and comprise the following sites: Fort Liberty, North Carolina; Fort Leonard Wood, Missouri; Fort Campbell, Kentucky; Fort Knox, Kentucky; Marine Corps Base Quantico, Virginia; Marine Corps Base Camp Lejeune, North Carolina; Shaw Air Force Base, South Carolina; Little Rock Air Force Base, Arkansas; Naval Station Norfolk, Virginia; and Naval Station Newport, Rhode Island (Figure 1) [21,22].

Samples were provided in a ratio of 1200 Soldiers, 600 Airmen, 600 Sailors, and 600 Marines. Inclusion additionally required the availability of two serum samples while stationed at the same military installation. Samples were separated by at least three years and up to four years apart. The provided serum was collected in the DoDSR between the years 2002 and 2010. The study excluded Reserve and National Guard service members on active duty.

Demographic data on de-identified subjects was reported from the DoDSR. Data included age in years, sex, self-reported race and ethnicity, rank, branch of service, occupation, and installation of assignment at the time of the serum draw. Rank was grouped as junior enlisted, senior enlisted, junior officer, warrant officers, and others. The military branch of service was indicated as Air Force, Army, Marine Corps, or Navy, and the installation location was reported as the individual’s location at the time the sera were obtained. Military occupations were grouped into categories, based on the likely risk of outdoor exposure to ticks: administrative, artillery/ordinances, base support, construction and engineering, education and training, flight operations, infantry/law enforcement, and medical (Table A1). The administrative group was chosen as the reference group. As many of the sera were collected upon entry into the military, military occupational code was based on the military occupation code assigned to the member at the time of their second serum draw.

Total IgE and alpha-gal specific IgE were measured by ImmunoCAP. Alpha-gal-specific IgE was considered positive at a cut-off of greater than or equal to 0.1 kU/L [23]. Incident alpha-gal seroconversion was defined as an alpha-gal IgE level <0.1 kU/L at baseline that was ≥0.1 kU/L at the follow-up blood draw. While the threshold of serum alpha-gal IgE levels associated with clinical symptoms can vary, a previous study identified a level of >2.0 kU/L to have a positive predictive value of 95% for clinical symptoms in a patient cohort [24]. In the present study, we also employed a similar threshold of >2.0 kU/L to identify subjects likely to have developed significant clinical symptoms. Given all sera were de-identified, there was no follow-up questionnaire or questionnaire given at the time of screening to determine dietary habits and any associated food reactions.

All statistical analysis was conducted using SAS v9.4 (Cary, NC, USA). Frequencies with percentages and medians with interquartile ranges (IQRs) were calculated for all variables overall and by alpha-gal conversion status. Wilcoxon rank-sum and Kruskal–Wallis tests were used for comparisons of IgE levels by demographic category. Because subjects had different times between serum samples, incidence densities were calculated for each demographic group using the number who seroconverted together with person-time between the two samples collected. A generalized linear model with a Poisson distribution and a likelihood ratio type 3 analysis was performed to assess significant differences in incidence density with the variables sex, race and ethnicity, rank, occupational category, branch of military service, and military installation. The exact lived geography and specific exposures of the individual members were unable to be collected; therefore, occupation and installation were used as surrogates for these data. The resulting *p*-values provide evidence for the significant difference in incidence density for each categorical variable. Poisson regression was then also used to estimate the rate ratios (RRs) and 95% confidence intervals (95% CIs) of seroconversion for each of the variables. Due to the strong correlation between occupation, installation, and branch of service, three separate adjusted models were run, including only one of each of these exposures while adjusting for demographic variables. The Uniformed Services University Institutional Review Board reviewed and approved this project. This study was a retrospective analysis of de-identified secondary use of banked sera without direct contact with participants; the Uniformed Services University Institutional Review Board (protocol approval number DBS.2019.037) reviewed and approved this study with a waiver of consent according to the ethical guidelines described in the United States Title 45 code of federal regulations 46.102(d).

## 3. Results

**Figure 2:**
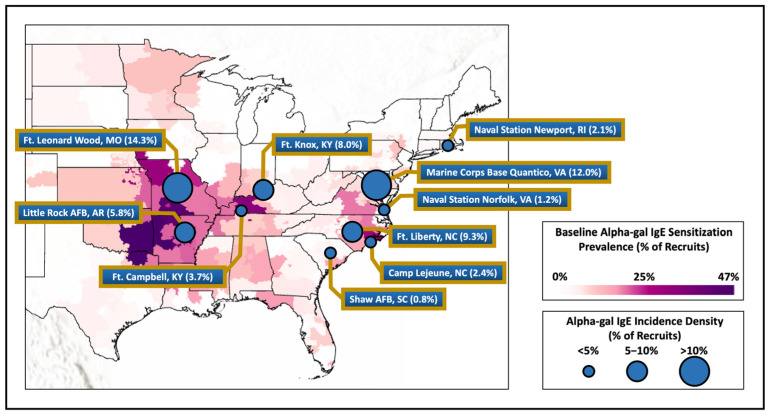
Baseline alpha-gal IgE sensitization of military members compared to measured incidence density of recruits from various installations around the United States. Legend. Map showing alpha-gal IgE sensitization at baseline laboratory testing for military recruits based on member’s home zip code of record at accession compared with the incidence density of alpha-gal IgE at the select military installations demonstrated by the circle size.

Of the 3000 subjects in the original study, all had their first and second serum samples successfully assayed and the median time between the first and second samples was 3.42 years (interquartile range [IQR], 3.19–3.68 years). Alpha-gal IgE was detected in 179 service members (6.0%) at the time of first assignment (baseline sample) to one of the selected military installations (Figure 1). The results of the analysis on these 179 military members who tested positive at baseline have been previously described and were excluded from further reporting or analysis, though we note that 31 (17.3%) of these had an increase in their alpha-gal IgE at the second serum sample (Figure 2) [22].

Among the 2821 who were negative for alpha-gal IgE at the baseline serum analysis, the median age was 19 (IQR 18–22), the majority were male (81.4%) and self-identified as White non-Hispanic (64.2%), and most were of junior enlisted rank (94.7%; Table 1).

As was defined by the study design, a majority of subjects were Army service members (40%) with about 20% equally in the Air Force, Marines, and Navy. The administrative (23%), infantry/law enforcement (22.3%), and flight operations (22.7%) occupational groups were the most common, followed by construction and engineering (12.6%) artillery/ordinance (7.4%), medical (5.5%), base support (5.1%), and education/training (1.3%).

Alpha-gal IgE seroconversion occurred in 138 service members (4.9%; Table 2).

**Figure 3:**
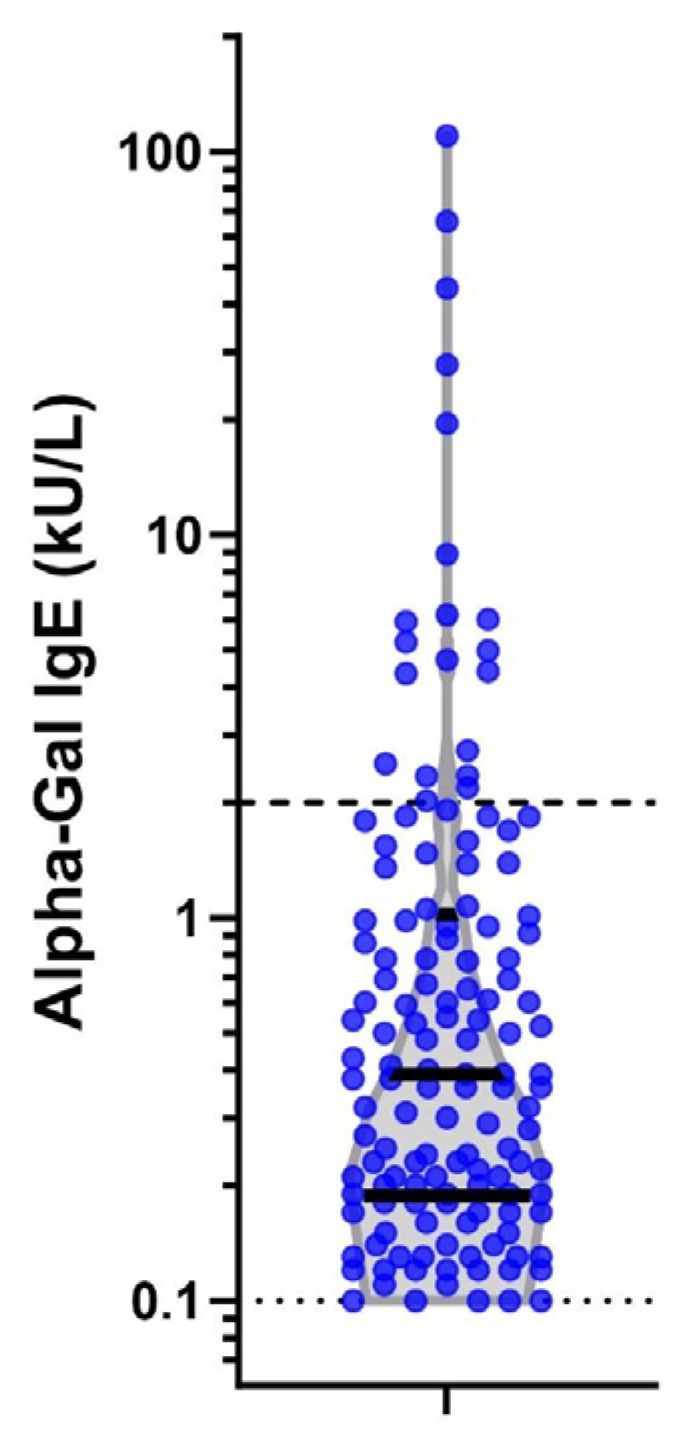
Alpha-gal IgE levels at second time point in 138 seropositive individuals. Legend: Violin plot on log scale of alpha-gal IgE levels among the 138 positive subjects at the second serum sample. Dark solid lines represent interquartiles of the data. The light dashed lines mark the diagnostic cut-off of the assay (0.1kU/L) and a level of 2 kU/L which has a stronger correlation with clinically symptomatic mammalian meat allergy.

The median alpha-gal IgE level for those who seroconverted was 0.39 kU/L (IQR 0.19–1.01; Figure 3). There were differences in levels of alpha-gal IgE by sex and race and ethnicity but not rank, base, branch of service, or occupation (Table A1). Seroconversion frequency and incident density were over three times higher in men than women (*p* < 0.001). Differences in race and ethnicity were evident, with seroconversion less common in Black (1.0%) and Hispanic (1.5%) personnel than White (6.6%) or Asian/Pacific Islander (7.1%) personnel (*p* < 0.001). There was no difference in seroconversion by rank (*p* = 0.149). The Army had the highest proportion of seroconversion (8.3%), then Air Force (3.7%), Marines (2.9%), and Navy (1.2%) (*p* < 0.001). There were significant differences in seroconversion between military installations (*p* < 0.001), with the highest absolute number of cases at Fort Liberty, NC, USA (*n* = 76; 9.3%), although the largest percentage of members who seroconverted were at Fort Leonard Wood, MO, USA (14.3%), followed by Marine Corps Base Quantico, VA, USA (12.0%). Seroconversion was most common in the infantry/law enforcement occupational category (12.7%) and lowest in the administrative category (1.2%; *p* < 0.001).

Seroconversion rate ratios were calculated and unadjusted RRs aligned closely with the results of seroconversion frequency and incident density (Table 3).

In the unadjusted analysis, females had a lower rate of seroconversion (RR, 0.34 95%; CI, 0.18–0.65). Compared to White personnel, Black (RR, 0.15; 95% CI, 0.05–0.40) and Hispanic (RR, 0.23; 95% CI, 0.10–0.57) personnel had lower rates of seroconversion. Personnel working in construction and engineering (RR, 3.00; 95% CI, 1.24–7.23) and infantry/law enforcement (RR, 10.43; 95% CI, 5.04–21.58) had higher rates than personnel in administration. Rates were highest at Fort Leonard Wood, MO, USA, and Marine Corps Base Quantico, VA, USA, as compared to Shaw Air Force Base, SC, USA. Unadjusted and adjusted rate ratios for models that included demographics and occupations as well as demographics and military installation are presented in Table 3. Adjusted and unadjusted rate ratios for specific branch of military service are presented in Table A3. In the adjusted analysis, differences in race and ethnicity, but not age, sex, or rank, persisted in the fully adjusted models. The higher rate of seroconversion among personnel in construction and engineering and infantry/law enforcement also persisted, as did differences between the military installations.

Finally, we evaluated the incidence of alpha-gal seroconversion to a level >2.0 kU/L, as this has been reported to have a 95% probability of clinically symptomatic AGS [24]. Of the 20 (0.7%) subjects who seroconverted to a level greater than 2.0 k/UL, 19 were male, 18 were White, all were junior enlisted, 17 were working infantry or law enforcement, and 14 were located at Fort Liberty, NC, USA (see Figure 3).

The full incidence density for the threshold of 2.0 k/UL is presented in Table A4.

## 4. Discussion

In this longitudinal historical investigation of US active-duty military members who were stationed in the range of *A. americanum*, we found an overall incidence of alpha-gal IgE seroconversion of 4.9 percent, with significant differences that related to military installation but also demographic factors. Installations with the highest rates of seroconversion generally aligned with recent maps outlining areas of the country with high rates of reported AGS or alpha-gal sensitization, including a previously reported map generated with baseline alpha-gal IgE data from this same cohort (see Figure 2) [8,9,22].

Seroconversion was highest among males, personnel identifying as White or Asian/Pacific Islander, and occupations involving construction and engineering or infantry/law enforcement. Differences in seroconversion by race and ethnicity, but not sex, persisted in adjusted models that accounted for age, sex, rank, and occupation.

This study is consistent with prior research showing that outdoor jobs and activities were associated with higher prevalence of alpha-gal IgE among those who live and/or work in this region. Investigating 52 state park and forestry employees from NC who worked outdoors and had frequent tick exposure, Mitchell et al. reported that 58% were sensitized to alpha-gal at study entry [25]. Bellamy et al. studied 46 forest workers from KY with significant tick bite history and reported that 40% had alpha-gal IgE ≥ 0.1 kU/L [26]. Investigations of patients in an internal medicine clinic in North Carolina revealed that alpha-gal sensitization was associated with three or more tick bites in the past year and spending 25 h per week or more outdoors [27]. A prior analysis of the current cohort by Ailsworth et al. that assessed baseline alpha-gal IgE in relation to home of record convincingly showed correlation between alpha-gal sensitization and *A. americanum* [22]. Studies outside of the USA also consistently show that outdoor exposure and tick bites are linked with alpha-gal sensitization [28,29,30,31,32]. Using a longitudinal sampling design, here we found that having an occupation with a presumed higher outdoor exposure (infantry/law enforcement or construction and engineering) demonstrated a greater risk of alpha-gal seroconversion as compared to occupations that were dominantly indoor (i.e., the administrative group). Of note, the three installations with the highest rates of incident alpha-gal IgE—Fort Leonard Wood, MO, Marine Corps Base Quantico, VA, and Fort Liberty, NC—were all in areas where *A. americanum* has been evaluated for many years and exposure to ticks would be anticipated. Seroconversion was notably low at both Navy installations, as could be anticipated due to the lack of exposure to wooded areas if the sailors were serving at sea for all or part of their assignment at these installations.

The impact of demographic factors such as sex and race and ethnicity on the development of alpha-gal sensitization is not clear. Some, but not all, studies have found that alpha-gal sensitization AGS is more common in males than females [13]. Gonzalez-Quintana et al. carried out a cross-sectional study of adults in Denmark (*n* = 2297) and Spain (*n* = 444) and found a higher frequency of alpha-gal sensitization in males than females in Denmark, but not Spain [29]. Westman et al. investigated 2201 young adults in the Swedish BAMSE birth cohort and found that alpha-gal sensitization was nearly 3-fold more frequent among males (8.9%) as females (3.4%) [13]. An analysis of baseline alpha-gal IgE among the 3000 military personnel in the current cohort revealed similar findings, with alpha-gal sensitization in 6.6% of the males and 3.3% of the females. The explanation for differences in sensitization between men and women could reflect innate biologic differences, but the current data suggest that differences more likely relate to an occupational and recreational risk of tick bites. Although alpha-gal seroconversion was ~3-fold more common among men (5.5%) than women (1.9%), this difference was not significant in models that adjusted for age, race and ethnicity, rank, and occupational category.

Few studies have systematically addressed alpha-gal sensitization related to race and ethnicity. Case–control studies by Kersh et al. and Wilson et al. both found that there were fewer Black individuals among AGS cases than controls, but both studies had limitations for interpreting differences by race and ethnicity [27,33]. The analysis of baseline alpha-gal IgE prevalence in the current cohort revealed lower frequency in Black (2.6%) versus White (7.5%) military members [22]. This difference persisted in models that adjusted for age, sex, home state of residence, and urban/rural status, but was limited by a lack of information about the occupational or recreational risk of tick bites. Interestingly, the current study accounts for occupational risk but rates of seroconversion were nonetheless lower in Black personnel as compared to White. It is possible that differences in ABO blood groups could explain some of this difference given that B-blood group is more common among Black individuals and is partially protective against the development of AGS, but further research is needed [33,34,35]. Regardless of the explanation, this finding is interesting in view of the fact that Black race and ethnicity is associated with higher rates for many common food allergies compared to those of White race and ethnicity [36,37].

This work provides longitudinal insight as to the incidence and seroconversion rates in persons living within the range of *A. americanum.* While the study population comprises a relatively young and healthy military population, it is unique in that it included a multitude of occupations, all branches of service, and provided evidence of seroconversion happening over time. Limitations of this study include the fact that the number of military members was not equally distributed among all installations, resulting in more military members coming from certain installations than others. The observed population was predominantly male, which may not allow for adequate comparisons in incidence density differences by sex. This study did not review available medical records of these personnel and therefore was not able to confirm the clinical significance of those with positive alpha-gal IgE. However, we did find that 20 (0.7 percent) of service members developed levels suggestive of significant clinical symptoms. At large military installations such as Fort Liberty, NC, with greater than 50,000 assigned service members, clinically significant alpha-gal seroconversion could be extrapolated to be as high as 350. There was no qualitative analysis of recreational activities that might put members at higher risk of acquiring AGS or confirmation of the activities required for members of certain occupations that may put them at higher risk of exposure to *A. americanum*. This study evaluates a period from 2002 to 2010, and shifts in the range of *A. americanum* have been reported since that time [9,22]. It is possible that the incidence seen in this study may not be reflective of the current geographical risks [38,39].

As an additional note, the seroconversion rates seen in this population may not be generalizable to the US population at large because of tick prevention measures utilized by the military that are not universally used by civilian populations. For example, military tick bite prevention measures primarily concern the avoidance of ticks and can include the wearing of long sleeved clothing and tucked pants, the use of products containing N,N-diethyl-meta-toluamide (DEET), and tick checks to include prompt removal if any are identified [40]. Since the 1930s, the US military has treated fabrics, including military uniforms, with insect repellents and starting in the 1990s, the military specifically began applying permethrin to uniforms, and it is regularly pre-applied to uniforms that are issued to recruits today [41]. Despite these measures, tick exposure is occurring in this population, although rates may be lower than would be seen in the general population with similar outdoor exposures living in these geographical locations.

## 5. Conclusions

In conclusion, the seroconversion of alpha-gal IgE is occurring in those who live within the range of *A. americanum* and is an occupational risk for members who have higher routine outdoor exposures, consistent with a causal role for tick bites in sensitization. US medical personnel should be aware of the possibility of sensitization to alpha-gal and the potential for the subsequent development of AGS in order to properly test and diagnose persons with consistent symptoms and a history of outdoor exposure in the range of *A. americanum* [42]. The universal adoption of tick exposure mitigation measures may limit sensitization to alpha-gal and be expected to prevent AGS. Further study should explore the influence of race and ethnicity on alpha-gal sensitization, evaluate the long-term effects and prognosis of AGS, and identify ways to adequately prevent and treat the syndrome.

## Figures and Tables

**Table 1:** Population demographics of the military cohort and members who were negative for alpha-gal IgE at baseline.

Characteristic	Total Cohort at Baseline (Percentage Within Each Category)	Subjects Who Were Negative for Alpha-Gal IgE at Baseline (Percentage Within Each Category)
Total	3000	2821
Median age in years (IQR)	19 (18–22)	19 (18–22)
Sex		
Male	2456 (81.9%)	2295 (81.4%)
Female	544 (18.1%)	526 (18.7%)
Race and Ethnicity		
White	1957 (65.2%)	1810 (64.2%)
Black	424 (14.1%)	413 (14.6%)
Hispanic	336 (11.2%)	328 (11.6%)
Native American	45 (1.5%)	44 (1.6%)
Asian/Pacific Islander	90 (3.0%)	85 (3.0%)
Other/Unknown	148 (4.9%)	141 (5.0%)
Rank		
Junior Enlisted	2840 (94.7%)	2670 (94.7%)
Senior Enlisted	21 (0.7%)	21 (0.7%)
Junior Officer	115 (3.8%)	107 (3.8%)
Other	24 (0.8)	23 (0.8%)
Occupational Category		
Administrative	690 (23.0%)	650 (23.0%)
Artillery/Ordinances	222 (7.4%)	209 (7.4%)
Base Support	153 (5.1%)	145 (5.1%)
Construction and Engineering	379 (12.6%)	356 (12.6%)
Education and Training	40 (1.3%)	36 (1.3%)
Flight Operations	681 (22.7%)	641 (22.7%)
Infantry/Law Enforcement	671 (22.4%)	629 (22.3%)
Medical	164 (5.5%)	155 (5.5%)
Branch		
Air Force	600 (20%)	567 (20.1%)
Army	1200 (40%)	1131 (40.1%)
Marines	600 (20%)	557 (19.7%)
Navy	600 (20%)	566 (20.1%)
Installation		
Fort Liberty, NC, USA	871 (29%)	821 (29.1%)
Fort Leonard Wood, MO, USA	44 (1.5%)	42 (1.5%)
Fort Campbell, KY, USA	230 (7.7%)	218 (7.7%)
Fort Knox, KY, USA	55 (1.8%)	50 (1.8%)
Marine Corps Base Quantico, VA, USA	26 (0.9%)	25 (0.9%)
Camp Lejeune, NC, USA	574 (19.1%)	532 (18.9%)
Shaw Air Force Base, SC, USA	252 (8.4%)	239 (8.5%)
Little Rock Air Force Base, AR, USA	348 (11.6%)	328 (11.6%)
Naval Station Norfolk, VA, USA	552 (18.4%)	519 (18.4%)
Naval Station Newport, RI, USA	48 (1.6%)	47 (1.7%)

**Table 2:** Population demographics by seroconversion incidence status.

	Seroconversion N and Percentage Within the Same Category That Seroconverted *N* = 138	Total Person-Years	Seroconversion Incidence Density (Per 1000 Person-Years)	*p* Value *
Sex				<0.001
Male	128 (5.6%)	7908.1	16.2	
Female	10 (1.9%)	1813.5	5.5	
Race and Ethnicity				<0.001
White	119 (6.6%)	6234.8	19.1	
Black	4 (1.0%)	1427.3	2.8	
Hispanic	5 (1.5%)	1126.0	4.4	
Native American	1 (2.3%)	155.7	6.4	
Asian/Pacific Islander	6 (7.1%)	292.0	20.5	
Other/Unknown	3 (2.1%)	485.9	6.2	
Rank				0.149
Junior Enlisted	132 (4.9%)	9200.9	14.3	
Senior Enlisted	3 (14.3%)	69.9	42.9	
Junior Officer	2 (1.9%)	372.6	5.4	
Other	1 (4.4%)	78.4	12.8	
Occupational Category				<0.001
Administrative	8 (1.2%)	2246.0	3.6	
Artillery/Ordinances	6 (2.9%)	714.5	8.4	
Base Support	4 (2.8%)	487.1	8.2	
Construction and Engineering	13 (3.7%)	1218.2	10.7	
Education and Training	2 (5.6%)	122.8	16.3	
Flight Operations	21 (3.3%)	2235.0	9.4	
Infantry/Law Enforcement	80 (12.7%)	2153.2	37.2	
Medical	4 (2.6%)	544.9	7.3	
Branch				<0.001
Air Force	21 (3.7%)	1972.3	10.6	
Army	94 (8.3%)	3876.4	24.2	
Marines	16 (2.9%)	1901.5	8.4	
Navy	7 (1.2%)	1971.6	3.6	
Installation				<0.001
Fort Liberty, NC, USA	76 (9.3%)	2827.1	26.9	
Fort Leonard Wood, MO, USA	6 (14.3%)	139.0	43.2	
Fort Campbell, KY, USA	8 (3.7%)	734.2	10.9	
Fort Knox, KY, USA	4 (8.0%)	176.1	22.7	
Marine Corps Base Quantico, VA, USA	3 (12.0%)	84.9	35.3	
Camp Lejeune, NC, USA	13 (2.4%)	1816.6	7.2	
Shaw Air Force Base, SC, USA	2 (0.8%)	836.4	2.4	
Little Rock Air Force Base, AR, USA	19 (5.8%)	1135.9	16.7	
Naval Station Norfolk, VA, USA	6 (1.2%)	1806.4	3.3	
Naval Station Newport, RI, USA	1 (2.1%)	165.1	6.1	

* *p* values were calculated using unadjusted type 3 analyses from the Poisson regression to compare the differences in incidence density by each demographic variable.

**Table 3:** Unadjusted and adjusted rate ratios including demographics, occupation, and military installation for alpha-gal IgE seroconversion.

	Unadjusted Rate Ratio	Model 1Adjusted Rate Ratio	Model 2Adjusted Rate Ratio
	(95% CI)	(95% CI)	(95% CI)
Age ^a^	1.05 (1.01–1.09)	1.04 (0.98–1.09)	1.03 (0.98–1.09)
Sex			
Male	ref	ref	ref
Female	0.34 (0.18–0.65)	0.82 (0.41–1.63)	0.66 (.34–1.29)
Race and Ethnicity			
White	ref	ref	ref
Black	0.15 (0.05–0.40)	0.26 (0.09–0.72)	0.19 (0.07–0.52)
Hispanic	0.23 (0.10–0.57)	0.34 (0.14–0.83)	0.31 (0.12–0.75)
Native American	0.34 (0.05–2.41)	0.48 (0.07–3.51)	0.52 (0.07–3.75)
Asian/Pacific Islander	1.08 (0.47–2.44)	1.54 (0.67–3.53)	1.28 (0.56–2.91)
Other/Unknown	0.32 (0.10–1.02)	0.54 (0.17–1.73)	0.47 (0.15–1.48)
Rank			
Junior Officer	ref	ref	ref
Junior Enlisted	2.67 (0.66–10.80)	2.24 (0.51–9.90)	3.68 (0.87–15.59)
Senior Enlisted	8.00 (1.34–47.86)	2.90 (0.44–19.32)	4.21 (0.67–26.41)
Other	2.38 (0.22–26.21)	2.29 (0.20–26.43)	3.63 (0.32–41.36)
Occupational Category			
Administrative	ref	ref	-
Artillery/Ordinances	2.36 (0.82–6.79)	1.85 (0.64–5.41)	-
Base Support	2.31 (0.69–7.66)	2.34 (0.70–7.82)	-
Construction and Engineering	3.00 (1.24–7.23)	2.46 (1.01–6.03)	-
Education and Training	4.57 (0.97–21.54)	4.06 (0.86–19.27)	-
Flight Operations	2.64 (1.17–5.96)	2.27 (0.998–5.16)	-
Infantry/Law Enforcement	10.43 (5.04–21.58)	7.05 (3.31–15.02)	-
Medical	2.06 (0.62–6.84)	2.20 (0.64–7.55)	-
Military Installation			
Shaw Air Force Base, SC, USA	ref	-	ref
Little Rock Air Force Base, AR, USA	7.00 (1.63–30.03)	-	6.64 (1.54–28.55)
Fort Liberty, NC, USA	11.24 (2.76–45.77)	-	8.18 (1.99–33.61)
Fort Leonard Wood, MO, USA	18.05 (3.64–89.42)	-	17.07 (3.43–84.87)
Fort Campbell, KY, USA	4.56 (0.97–21.46)	-	3.95 (0.83–18.71)
Fort Knox, KY, USA	9.51 (1.74–51.87)	-	7.41 (1.35–40.69)
Marine Corps Base Quantico, VA, USA	14.78 (2.47–88.45)	-	14.01 (2.32–84.54)
Camp Lejeune, NC, USA	2.99 (0.68–13.26)	-	2.71 (0.61–12.06)
Naval Station Norfolk, VA, USA	1.39 (0.28–6.88)	-	1.38 (0.28–6.86)
Naval Station Newport, RI, USA	2.53 (0.23–27.93)	-	2.47 (0.22–27.37)

Note: To address the influence of multicollinearity between military installation and occupation, two distinct models are analyzed. This allows for a clearer interpretation of the independent effects of each variable. Both models exclusively use the variables listed in the table. ^a^. Rate ratio represents one year increase in age.

## Data Availability

The data related to this study will be made available on request from the corresponding author.

## Appendix A

**Table A1:** Categories of military occupations.

Occupational Category	Occupation Title
Administrative	General Administration, Automated Data Progressing (ADP) Computers, Analysis, Auditing and Accounting, Aviation Maintenance Records and Reports, Chaplain, Chaplain’s Assistant, Combined Personnel and Administration, Comptroller and Fiscal Disbursing, Illustrating, Image Interpretation, Information and General Education, Intelligence, Intercept Operator, Interior Communications, Language Interrogation/Interpretation, Legal, Lithography, Manpower and Personnel, Meteorologist and Weather, Musician, General Operational Intelligence, Operations Staff, Operators/Analyst, Photography, Personnel, Procurement and Production, Recruiting and Counseling, Supply Administration
Artillery/Ordinances	Ammunition Repair, Armor and Amphibious, Artillery and Gunnery, Aviation Ordnance, Chemical, Explosive Ordnance Disposal/Underwater Demolition Team EOD/UDT, Ground and Naval Arms, Main Propulsion, Missile Artillery Operating Crew, Missile Fuel and Petroleum, Nuclear, Biological, and Chemical Warfare Specialist, Ordnance, Rocket Artillery, Small Arms Repair
Base Support	Food Service, Laundry and Personal Service, Logistics, Postal, Supply, Utilities, Warehousing and Equipment Handling, Sales Store, Teletype and Cryptographic, Equipment
Construction and Engineering	Automotive, Auxiliaries, Boatswain, Combat Engineering, Combat Operations Control, Construction and Utilities, Construction Equipment, Construction Equipment Operation, General Construction, Electric Power, Electrical/Electronic, Electricians, Electronic Instruments, Fabric, Leather and Rubber, Lineman, Machinist, Metal Body Repair, Motor Vehicle Operators, Other Mechanical and Electrical Equipment, Seamanship, Surveying, Tracked Vehicles Transportation, Welding, Woodworking
Education and Training	Cadets and Other Officer Candidates, Students, Military Training Instructor, Non-occupational Other, Not Occupationally Qualified, Undesignated Occupations
Flight Operations	Air Crew, Air Traffic Control, Air Traffic Control Radar, Aircraft Accessories, Aircraft Engines, Aircraft Launch Equipment, Aircraft Structures, General Aircraft, Aviation Maintenance and Allied, Communications and Radar, Communications Radio, Firefighting and Damage Control, Flight Operations, Forward Area Equipment Support, Helicopter Pilot, Navigation, Communications and Countermeasure, Non-Code Radio, Radar, Radio Code
Infantry/Law Enforcement	Corrections, Infantry, Investigation, Law Enforcement, Police, Security Guards, Small Arms Repair, Special Forces
Medical	Behavioral Sciences/Mental Health Services, Bioenvironmental Engineering, Biomedical Laboratory Services, Comprehensive Dentistry, Dental Care, Diet Therapy, Emergency Medicine, Endodontics, Environmental Health Services, Environmental Health/Preventive Medicine Services, Expeditionary Medical Services, Family Practice, Functional Analysis, Gastroenterology, General Dentistry, Nursing, General Surgery, Health Services Administration, Internal Medicine, Medical Administration, Medical Care and Treatment, Medical Logistics, Medical/Surgical Nurse, Operating Room Services, Oral Maxillofacial Surgery, Pharmacy, Physical and Occupational Therapy, Physiology, Diagnostic Radiology, Social Scientists, Veterinarian and Veterinary Medicine

**Table A2:** Median and interquartile range of alpha-gal IgE levels by demographic and occupational categories among seroconverted subjects.

	Seroconversion Number	Alpha Gal IgE (kU/L [IQR])	*p*-Value
All Combined	138	0.39 (0.19–1.01)	
Sex			0.02 ^a^
Male	128	0.40 (0.20–1.06)	
Female	10	0.17 (0.13–0.41)	
Race and Ethnicity			0.02 ^b^
White	119	0.48 (0.21–1.07)	
Black	4	0.12 (0.11–0.17)	
Hispanic	5	0.41 (0.15–1.91)	
Native American	1	0.91 (0.91–0.91)	
Asian/Pacific Islander	6	0.13 (0.12–0.14)	
Other/Unknown	3	0.31 (0.19–0.38)	
Rank			0.33 ^b^
Junior Enlisted	132	0.39 (0.19–1.06)	
Senior Enlisted	3	0.14 (0.14–0.54)	
Junior Officer	2	0.19 (0.13–0.25)	
Other	1	0.91 (0.91–0.91)	
Base			0.52 ^b^
Camp Lejeune, NC	13	0.36 (0.21–0.48)	
Fort Liberty, NC	76	0.53 (0.21–1.43)	
Fort Campbell, KY	8	0.34 (0.14–0.55)	
Fort Knox, KY	4	0.25 (0.18–0.82)	
Fort Leonard Wood, MO	6	0.46 (0.14–2.02)	
Little Rock Air Force Base, AR	19	0.27 (0.13–1.79)	
Naval Station Newport, RI	1	0.53 (0.53–0.53)	
Naval Station Norfolk, VA	6	0.24 (0.13–0.55)	
Marine Corps Base Quantico, VA	3	0.98 (0.14–19.50)	
Shaw Air Force Base, SC	2	0.19 (0.19–0.19)	
Occupational Category			0.13 ^b^
Administrative	8	0.27 (0.12–0.54)	
Artillery/Ordinances	3	1.05 (0.36–1.83)	
Base Support	4	0.31 (0.16–0.51)	
Construction and Engineering	16	0.24 (0.16–0.54)	
Education and Training	2	0.36 (0.12–0.60)	
Flight Operations	21	0.21 (0.14–0.78)	
Infantry/Law Enforcement	80	0.53 (0.22–1.51)	
Medical	4	0.26 (0.19–1.03)	
Service			0.41 ^b^
Army	94	0.49 (0.19–1.38)	
Marines	16	0.39 (0.19–0.60)	
Air Force	21	0.24 (0.19–0.78)	
Navy	7	0.30 (0.13–0.55)	

^a^. Wilcoxon rank-sum test; ^b^. Kruskal–Wallis test. Abbreviations: IgE, Immunoglobulin E; IQR, Interquartile Range; kU/L, kilounits per liter.

**Table A3:** Unadjusted and adjusted rate ratios for branch of military service.

Branch	Unadjusted Rate Ratios	Adjusted Rate Ratios ^a^
	(95% CI)	(95% CI)
Navy	ref	ref
Air Force	3.00 (1.27–7.05)	2.94 (1.24–6.95)
Army	6.83 (3.17–14.72)	5.25 (2.41–11.44)
Marines	2.37 (0.98–5.76)	2.15 (0.88–5.27)

^a^. Adjusted for age, sex, race, and rank. Abbreviation: CI, Confidence Interval.

**Table A4:** Population demographics by seroconversion incidence status with alpha-gal IgE > 2.00 kU/L.

	Seroconversion N and Percentage Within the Same Category That Seroconverted *N* = 20	Total Person-Years	Seroconversion Incidence Density (Per 1000 Person-Years)	*p* Value *
Sex				<0.001
Male	19 (0.8%)	7908.1	2.40	
Female	1 (0.2%)	1813.5	0.55	
Race and Ethnicity				<0.001
White	18 (1.0%)	6234.8	2.89	
Black	0	1427.3	0	
Hispanic	1 (0.3%)	1126.0	0.89	
Native American	0	155.7	0	
Asian/Pacific Islander	1 (1.2%)	292.0	3.42	
Other/Unknown	0	485.9	0	
Rank				0.149
Junior Enlisted	20 (0.8%)	9200.9	2.17	
Senior Enlisted	0	69.9	0	
Junior Officer	0	372.6	0	
Other	0	78.4	0	
Occupational Category				<0.001
Administrative	0	2246.0	0	
Artillery/Ordinances	0	714.5	0	
Base Support	0	487.1	0	
Construction and Engineering	1 (0.3%)	1218.2	0.82	
Education and Training	0	122.8	0	
Flight Operations	2 (0.3%)	2235.0	0.89	
Infantry/Law Enforcement	17 (2.7%)	2153.2	7.90	
Medical	0	544.9	0	
Branch				<0.001
Air Force	2 (0.4%)	1972.3	1.01	
Army	17 (1.5%)	3876.4	4.39	
Marines	1 (0.2%)	1901.5	0.53	
Navy	0	1971.6	0	
Installation				<0.001
Fort Liberty, NC	14 (1.7%)	2827.1	4.95	
Fort Leonard Wood, MO	2 (4.8%)	139.0	14.39	
Fort Campbell, KY	1 (0.5%)	734.2	1.36	
Fort Knox, KY	0	176.1	0	
Marine Corps Base Quantico, VA	1 (4.0%)	84.9	11.78	
Camp Lejeune, NC	0	1816.6	0	
Shaw Air Force Base, AR	0	836.4	0	
Little Rock Air Force Base, AR	2 (0.6%)	1135.9	1.76	
Naval Station Norfolk, VA	0	1806.4	0	
Naval Station Newport, RI	0	165.1	0	

Abbreviations: IgE, Immunoglobulin E; kU/L, kilounits per liter. * *p* values were calculated using unadjusted type 3 analyses from the Poisson regression to compare the differences in incidence density by each demographic variable.
